# Aversive Bimodal Associations Differently Impact Visual and Olfactory Memory Performance in *Drosophila*

**DOI:** 10.1016/j.isci.2022.105485

**Published:** 2022-11-03

**Authors:** Devasena Thiagarajan, Franziska Eberl, Daniel Veit, Bill S. Hansson, Markus Knaden, Silke Sachse

**Affiliations:** 1 Research Group Olfactory Coding, Max Planck Institute for Chemical Ecology, Jena, Germany; 2 Department of Evolutionary Neuroethology, Max Planck Institute for Chemical Ecology, Jena, Germany; 3 Max Planck Institute for Chemical Ecology, Jena, Germany

**Keywords:** behavioral neuroscience, sensory neuroscience

## Abstract

Animals form sensory associations and store them as memories to guide behavioral decisions. Although unimodal learning has been studied extensively in insects, it is important to explore sensory cues in combination because most behaviors require multimodal inputs. In our study, we optimized the T-maze to employ both visual and olfactory cues in a classical aversive learning paradigm in *Drosophila melanogaster*. In contrast to unimodal training, bimodal training evoked a significant short-term visual memory after a single training trial. Interestingly, the same protocol did not enhance short-term olfactory memory and even had a negative impact. However, compromised long-lasting olfactory memory significantly improved after bimodal training. Our study demonstrates that the effect of bimodal integration on learning is not always beneficial and is conditional upon the formed memory strengths. We postulate that flies utilize information on a need-to basis: bimodal training augments weakly formed memories while stronger associations are impacted differently.

## Highlights

• Aversive olfactory memory is stronger and lasts longer than visual memory
• Bimodal training with odor and color selectively improves short-term visual memory
• Long-lasting, but not short-term, olfactory memory is enhanced by bimodal training
• The value of bimodal sensory integration depends on the formed memory strength

## Graphical abstract

**Figure:**
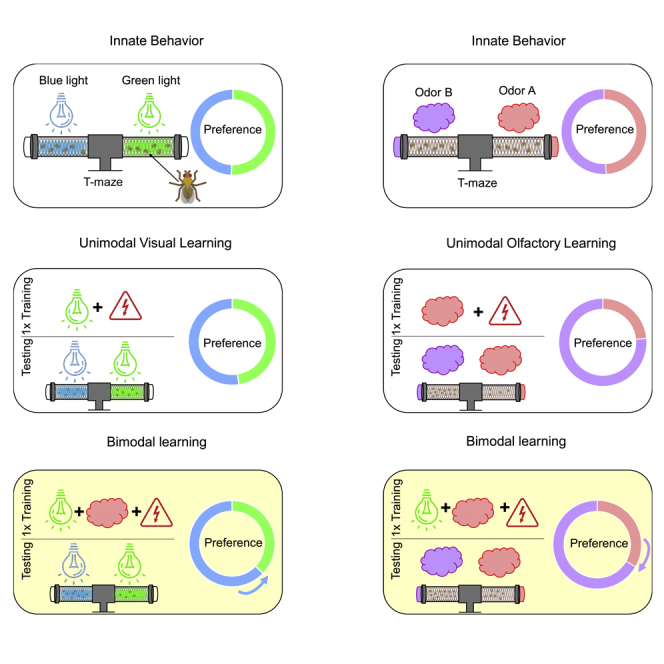

## Introduction

All organisms develop intricate sensory relationships with the environment around them. These interactions are essential to sustain and propagate life across diverse habitats. Insects, being one among the most versatile organisms to walk the Earth, depend enormously on these sensory interactions to perform a wide variety of behaviors, therefore providing strong research interests for entomologists and neurobiologists alike. The use of sensory signals to elicit foraging, courtship, mating, and predatory behaviors have been long studied and characterized across different insect orders.^1^^,^^2^^,^^3^^,^^4^^,^^5^^,^^6^^,^^7^^,^^8^^,^^9^^,^^10^ Although insects are equipped with dedicated unimodal sensory systems on their bodies and in the brain, signals available in nature are hardly isolated, and insects perform extensive multisensory consolidation before these signals become usable instructions for guiding a behavior. Integration of sensory information deriving from diverse sensory modalities occurs in specific brain centers. In vertebrates, the cortex and the superior colliculus are known to perform this function with the help of bimodal and trimodal neurons.^11^^,^^12^^,^^13^ In insects, different higher-order centers such as the mushroom body, the lateral horn, and the central complex have been shown to receive multimodal information from different sensory pathways.^14^^,^^15^^,^^16^^,^^17^

In different scenarios, sensory systems interact with and affect one another. Associations are often made using these sensory signals to remember potential outcomes in a specific situation. Both beneficial and adverse outcomes are learned and retained in the insect brain for either a short or a long period. Research in learning and memory spanning several decades has shown that insects can learn olfactory and visual signals, with members of some orders showing more acuity in this ability than others. Honeybees can learn to associate odors and several attributes of visual information such as shapes, colors, and patterns with potential rewards or punishments.^1^^,^^6^^,^^18^ Vinegar flies (*Drosophila melanogaster*), while investigated thoroughly for their remarkable olfactory learning ability,^19^^,^^20^ do not exhibit comparable strengths in color learning.^21^^,^^22^ Ants, on the other hand, learn both olfactory and visual cues in the process of foraging and nest identification but exhibit much faster learning rates when exposed to bimodal cues.^23^^,^^24^^,^^25^

Conventional olfactory associative conditioning experiments have been extensively investigated in *D. melanogaster* using a T-maze.^20^^,^^26^^,^^27^ A novel circular conditioning arena was developed to study color learning in flies,^22^^,^^28^ but tethered flight arenas are also commonly used to investigate both pattern learning and multisensory integration during flight behaviors.^29^^,^^30^^,^^31^ One of the first evidences of cross-modal information transfer in flies was also seen in a flight simulator, where bimodal training of poorly learned unimodal stimuli showed enhanced memory performances for both pattern and odor learning.^32^ A similar work in harnessed honeybees also highlighted the use of weak-intensity stimuli in bimodal training.^33^ These observations warrant an important question: Is the presence of an additional stimulus reinforcing the same outcome always beneficial in the process of learning or is it conditional upon the requirements of the insect?

In an attempt to answer this question, we aimed to dissect the effects of bimodal associative conditioning on the visual and olfactory memory of flies. Although studies on color vision in *D. melanogaster* have clearly revealed the ability of these flies to perceive and prefer different wavelengths,^34^^,^^35^^,^^36^ work done using color stimuli in associative conditioning experiments has not yielded very high learning performances. In fact, for aversive conditioning assays, flies require intense training trials for achieving high learning scores, and the acquired visual memory decays almost entirely after 6 h.^21^ These characteristics of color learning make it a suitable paradigm to be used as a component of bimodal training. In our work, we optimized the conventional Tully T-maze to present the flies with either unimodal or bimodal olfactory (odors) and visual stimuli (lights of different wavelengths) that were paired with an aversive reinforcement (electric shock). Using this approach, we demonstrate that bimodal integration has conditional and diverse effects on the learning and memory performances of different stimuli.

## Results

### Flies Exhibit Varied Learning Performances for Olfactory and Visual Stimuli

**Figure 1:**
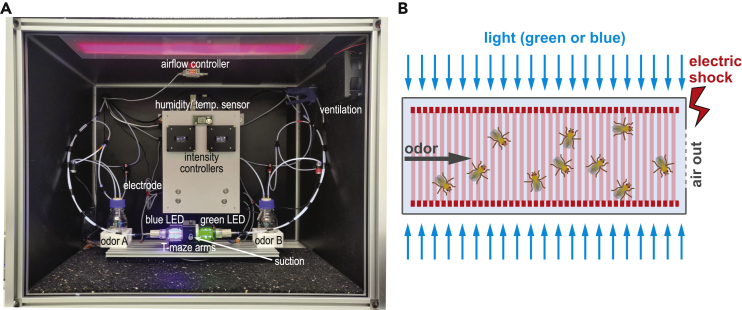
A Modified T-Maze to Study Unimodal Olfactory, Visual, and Bimodal Learning in *D. melanogaster* (A) Image of the T-maze design adapted and optimized from the conventional Tully T-maze apparatus with different labels indicating the components. The LED contraptions are kept open for illustration purposes only. (B) Schematic of the inside of the training tube during stimulus presentation.

In order to investigate the differences in unimodal and bimodal learning of vision and olfaction, we adapted and modified the conventional Tully T-maze (Figure 1A). Pulsated odor flow and homogeneous illumination from individual LED arrays were paired with the delivery of electric shock. This design allows for both synchronous and separated presentation of sensory stimuli, depending on the experimental requirements (Figure 1B). We used two food odors found in fruits or yeast that are attractive for the flies—acetoin acetate (AAC) and ethyl butyrate (ETB)^37^—as well as two wavelengths of light—blue (452 nm) and green (520 nm). These wavelengths are perceived by distinct photoreceptors^28^^,^^38^^,^^39^ and have been shown to be distinguishable in previous visual learning paradigms.^22^

**Figure 2:**
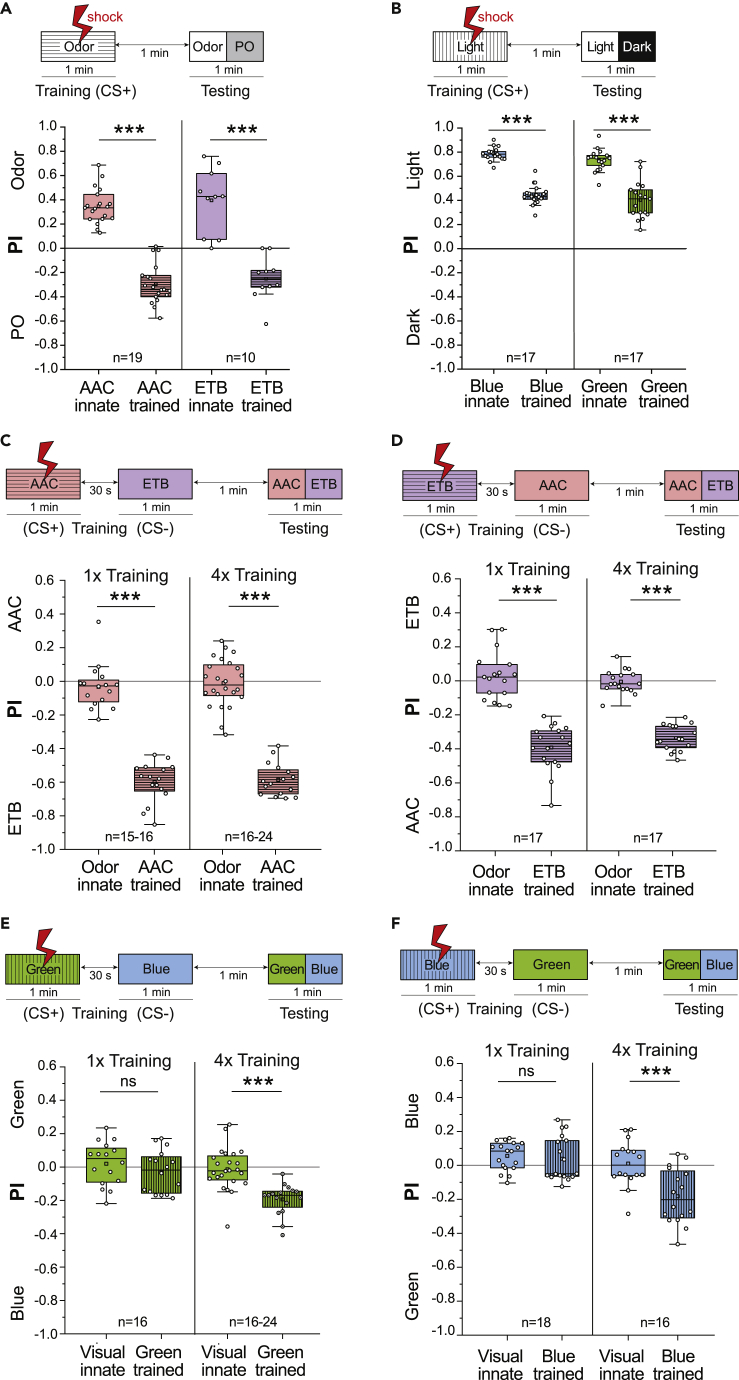
*D. melanogaster* Exhibits Varied Aversive Learning Responses for Different Odors and Colors (A) Training protocol for absolute olfactory conditioning. Preference indices for acetoin acetate (AAC) and ethyl butyrate (ETB) against paraffin oil (PO) are plotted for flies tested for innate behavior and for flies that were subjected to absolute aversive conditioning. Significant aversive olfactory memory was seen in flies for both AAC and ETB. (B) Training protocol for absolute visual conditioning. Preference indices for blue and green wavelengths are plotted for flies tested for innate behaviors and flies that were subjected to absolute aversive conditioning. Significant suppression of positive phototaxis, but no avoidance was seen for both blue and green wavelengths. (C and D) Training protocol for differential olfactory conditioning. Preference indices of flies tested for innate and learned behaviors for AAC and ETB are plotted. Both one-trial and four-trial (1x/4x) training were done. Aversive olfactory short-term memory was strong and similar between 1x and 4x training protocols. (E and F) Training protocol for differential visual conditioning. Preference indices of flies tested for innate and learned behaviors for green and blue wavelengths are plotted. Aversive visual short-term memory in flies is weak and was not seen in the 1x differential training protocol. When training trials were increased to four, a significant visual memory was observed. Each data point represents one experimental trial with 60–80 flies. Comparisons between two normally distributed groups were carried out using parametric unpaired two-sample Student's t test (∗∗∗p < 0.001).

We initially employed unimodal aversive conditioning to compare learning efficiencies of olfactory and visual cues. We established two kinds of training protocols in our modified T-maze setup. First, we used an absolute conditioning paradigm (Figures 2A and 2B), where we trained a group of flies to associate an odor or color (conditioned stimulus [CS]) with an electric shock of AC 90V (unconditioned stimulus [US]). In the testing phase, the flies were allowed to choose between the stimulus and the control, i.e., odor vs. solvent or the illuminated vs. the nonilluminated arm of the T-maze, in the absence of the reinforcement. Unlike previous protocols for visual learning,^22^ we did not present any electric shock to arouse the flies prior to the testing phase. In addition, we tested the innate preference to different stimuli in a separate group of flies for every experiment and calculated an innate preference index. The preference indices were used to determine differences in the behavior of flies that were conditioned and those that were not.

When presented with an absolute choice against the solvent paraffin oil (PO), the flies showed a significant innate attraction toward both the odors. In flies that were subjected to one-trial absolute aversive conditioning, this attraction shifted into significant avoidance, with the animals choosing the solvent arm consistently (Figure 2A). When tested for their choice between the illuminated and nonilluminated arms of the T-maze, flies showed high preferences to both blue and green wavelengths owing to a strong positive phototaxis response. In our experiments, we show that upon unimodal training of the light with an electric shock, flies exhibited a significant reduction in positive phototaxis (Figure 2B). Nonassociative effects were controlled for by training the flies with PO and darkness paired with an electric shock (Figures S1A and S1B). While the reduced positive phototaxis response indicates a successful reinforcement of the punishment, it is also evident from these results that the strength of the training is not sufficient to entirely overcome the innate behavior of the flies. Conceivably, positive phototaxis represents a strong innate driving force that cannot be easily counteracted.

As a second learning paradigm, we used a differential conditioning protocol (Figures 2C–2F), where flies were first trained to associate one odor or color with the electric shock (CS+), while a second odor or color was not paired with any reinforcement (CS-). After training, the choices between the two odors or colors were tested. For olfactory conditioning, the concentrations of the odors were standardized beforehand to abolish any innate bias, such that both odors were preferred equally by naive flies. Differentially trained flies exhibited a strong aversion to the CS+ odor and shifted their preference to the arm with the CS- odor (Figures 2C and 2D). We also observed that the one-trial differential training was sufficient to achieve the same strength of olfactory memory as a four-trial training with an intertraining interval (ITI) of 1 min, emphasizing the ability of flies to perform very efficient aversive olfactory learning tasks (Figures 2C and 2D). Interestingly, we also observed that flies showed odor-specific learning performances, with AAC retaining a stronger short-term memory than ETB (Figure S2A).

For differential visual conditioning, similar to the olfactory protocol, the intensities of the blue and green wavelengths were optimized to ensure equal preference toward both wavelengths, thereby circumventing the phototaxis bias that was observed in the absolute conditioning paradigm (Figure 2B). Naive flies were equally distributed in both the arms of the T-maze before training. Notably, flies subjected to one-trial differential training of visual stimuli did not show significant learning performances, but when the training cycle was repeated four times, they exhibited robust visual learning (Figures 2E and 2F). Put together, our results demonstrate that the same training paradigm results in varying strengths of aversive learning for olfactory and visual stimuli in flies (Figures 2, S2A, and S2B).

### Presence of Odor During Training Enhances Visual Learning in an Absolute Conditioning Paradigm

**Figure 3:**
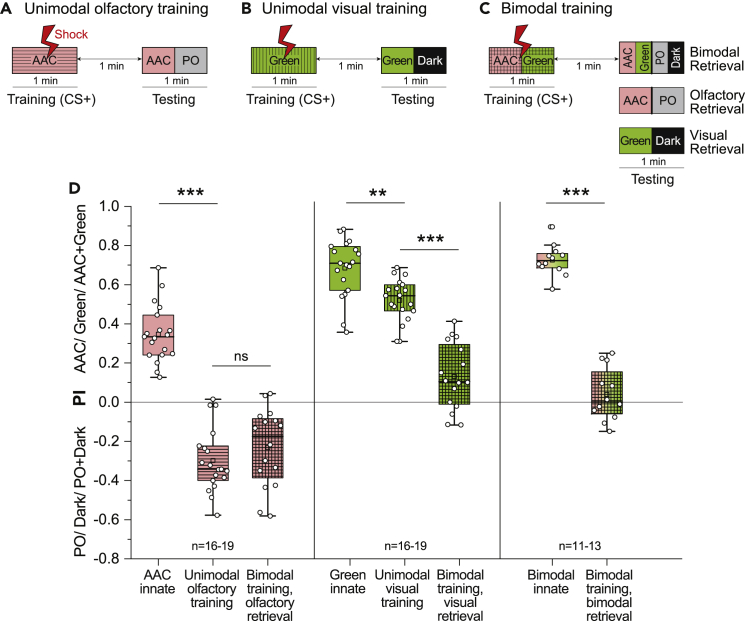
Presence of Odor During Absolute Conditioning Enhances Unimodal Visual Memory (A and B) Training protocol for absolute aversive unimodal olfactory and visual conditioning. (C) Training protocol for absolute aversive bimodal conditioning with three different testing protocols. (D) Innate and learned preference indices for AAC, green, and AAC + green are plotted. Visual memory retrieval after bimodal training shows stronger suppression of positive phototaxis as compared to only unimodal visual training. Olfactory memory retrieval after bimodal training does not differ significantly from only unimodal olfactory training. Statistical comparisons between more than three normally distributed datasets were made using one-way ANOVA followed by Bonferroni correction and Tukey’s post-hoc test (∗∗p < 0.01, ∗∗∗p < 0.001). Comparison between two normally distributed datasets was made using unpaired Student's t test with a Welch’s correction (∗∗∗p < 0.001).

After successfully establishing aversive unimodal olfactory and visual conditioning in our setup, we next aimed to dissect the effect of a bimodal training paradigm on the unimodal learning performance of flies. We first addressed the following question: Can a combination of two sensory modalities during associative training lead to a stronger learning efficiency? We delivered three different kinds of CS+: unimodal olfactory, unimodal visual, and a bimodal (odor + light) stimulus (Figures 3A–3C). Unimodally trained flies were tested only with the trained olfactory or visual stimulus, while bimodally trained flies were tested either with the composite bimodal stimulus or with the individual olfactory and visual components (Figure 3C). Notably, bimodal training of flies followed by bimodal testing yielded the same strength of memory as unimodal olfactory training (Figure 3D). However, it was difficult to conclude the individual contribution of each sensory modality in this composite memory performance, especially since an additive effect was not evident. We therefore turned our attention to the retrieval of olfactory and visual memory responses individually after bimodal training. When retrieved separately, the olfactory memory of bimodally trained flies was not significantly different from that of the flies that were trained unimodally. We hypothesize that this lack of enhancement could be attributed to the already strong olfactory memory, which cannot be enhanced further by the addition of a visual stimulus during training. Interestingly, when flies were trained bimodally, but tested unimodally with only the light stimulus, we observed a striking reduction in their positive phototaxis response, even more so than in their unimodally trained counterparts (Figure 3D). Hence, an improved visual memory was observed in comparison to unimodal training of the visual stimulus. In this experiment, the absence of the odor during the test phase negated any direct effect of the strongly learned odor response, and the flies retained only the visual memory following bimodal training. To further emphasize the effects of bimodal training, we calculated the difference between the preference indices of the innate and the learned behaviors for different training paradigms (Figure S2C). These results indicate that the presence of an odor along with a light stimulus leads to stronger acquisition of a visual memory.

### One-Trial Differential Bimodal Training of Colors Yields Significant Visual Memory

**Figure 4:**
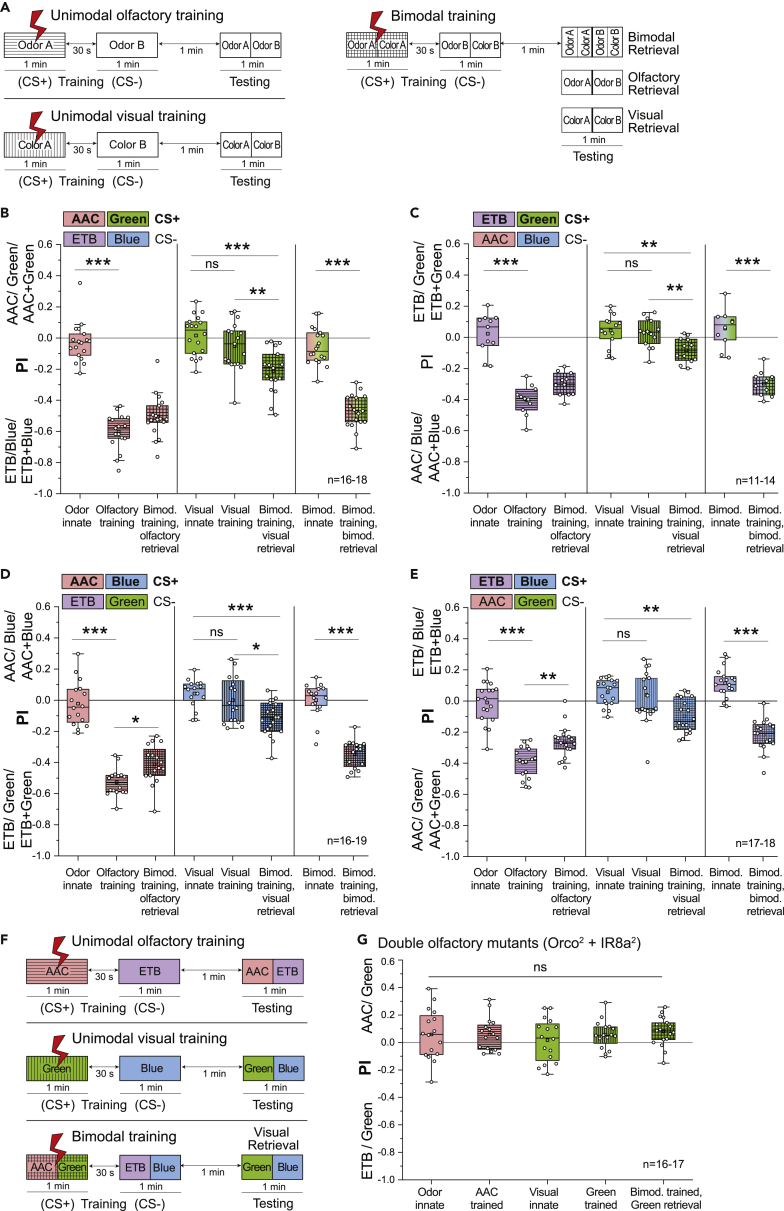
One-Trial Differential Bimodal Training of Colors Yields Significant Short-Term Visual Memory (A) General training protocols for differential unimodal olfactory, visual, and bimodal training along with corresponding testing procedure. (B–E) Innate and learned preference indices for unimodal and bimodal differential training, plotted for different combinations of odors and colors. Emergence of visual memory can be seen across combinations after bimodal training. A reduction of olfactory memory was also observed when flies were bimodally trained but unimodally retrieved. Composite bimodal memory was not stronger than unimodal olfactory memory. All statistical comparisons between normally distributed datasets were made using one-way ANOVA followed by Bonferroni correction and Tukey’s post-hoc test. Kruskal-Wallis test followed by Dunn-Bonferroni correction was done for comparisons between three or more groups that had nonparametric properties (∗p < 0.05, ∗∗p < 0.01, ∗∗∗p < 0.001). (F) General training protocols for differential unimodal olfactory, visual, and bimodal training along with corresponding testing procedure. (G) Preference indices of flies tested for innate and learned behaviors after unimodal and bimodal differential training for double olfactory mutants lacking *Orco* and *IR8a* co-receptors. Inability of the flies to detect odors abolishes olfactory learning and, therefore, does not show a significant visual memory after bimodal training. All statistical comparisons were made using one-way ANOVA followed by Bonferroni correction and Tukey’s post-hoc test (ns, not significant, p > 0.05).

We next turned to differential conditioning to test whether a similar enhancement of visual memory by bimodal training could also occur in another learning paradigm (Figure 4A). Separate groups of flies were used to monitor innate preferences for all experimental conditions, and they served as controls for comparison with flies that were subjected to the training paradigm. Bimodal training consisted of a combination of one odor and color (e.g., AAC + green) serving as CS+ and another combination serving as CS- (e.g., ETB + blue). Following bimodal training, testing was carried out in three different categories as described before to analyze (1) a composite bimodal memory, (2) an olfactory memory, and (3) a visual memory. We performed one-trial differential bimodal training (i.e., 1x) for all possible odor and color combinations. To deduce the effect of bimodal training only on the visual memory, we trained the flies to the bimodal stimulus but tested them afterward only to the light stimulus, excluding the effect of a learned odor bias (Figure 4A). We observed that a significant avoidance was displayed toward the trained color even in the absence of the paired odor during testing. The addition of odor information during the training procedure induced a significant visual memory to emerge, while training with only a unimodal visual stimulus did not yield any memory. This effect was evident across all color and odor combinations (Figures 4B–4E). Conversely, in order to identify the effect of bimodal training on only the olfactory memory, we trained the flies to the bimodal stimulus but tested them afterward only to the odor stimulus, excluding the effect of any color bias. Notably, in these experiments, we observed a reduction in the strength of the olfactory memory. In combinations involving the blue wavelength, the retrieved olfactory memory after bimodal training was significantly weaker than the unimodal olfactory memory (Figures 4D and 4E), while a trend of reduction was observed in combinations involving the green wavelength. In three combinations, the composite bimodal memory was significantly weaker than the unimodal olfactory memory (Figures 4C–4E).

In order to confirm the role of olfactory input in enhancing visual short-term memory, we performed the same experiments for one combination (i.e. AAC + green) with olfactory mutant flies that lack the odorant receptor (OR) co-receptor (*Orco*) and the ionotropic receptor (IR) co-receptor (*IR8a*) (Figures 4F and 4G). While *Orco* is co-expressed with ORs and is crucial for proper OR functioning, *IR8a* plays a role in IR-mediated acid and aldehyde sensing.^40^ Mutant flies lacking the expression of *Orco* and *IR8a* cannot detect the odors that were used in our assay and, therefore, did not show any significant olfactory memory. Subsequently, they also did not reveal any significant visual memory after being bimodally trained to both an odor and a visual stimulus (Figure 4G). These results emphasize the importance of an olfactory stimulus, especially the learned response to the odors, in the formation of a visual short-term memory after bimodal training. This observation further confirms that bimodal integration underlies the enhancement of visual memory.

### Bimodal Training Selectively Enhances Weakened Olfactory and Visual Memories

**Figure 5:**
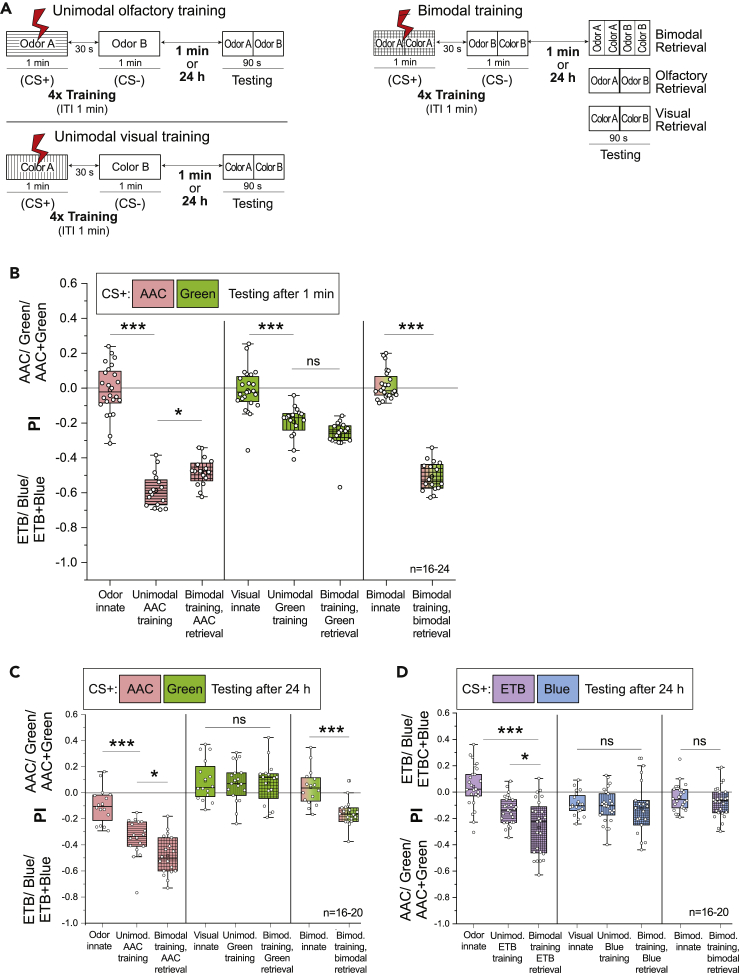
Bimodal Training Selectively Enhances Weakened Olfactory and Visual Memories (A) General massed four-trial (4x) training protocols, with an intertraining interval of 1 min, for differential unimodal olfactory, visual, and bimodal training along with corresponding testing protocols. Short-term testing was done 1 min after the last training cycle. For long-lasting memory, testing was done 24 h after the last training cycle. (B) Short-term memories retrieved after four-trial training plotted as changes in preference indices of trained and untrained flies to AAC, green, and AAC + green. Four training trials lead to significant short-term visual memory to form. This is not enhanced further when bimodally trained but unimodally retrieved. A reduction of olfactory memory was observed when bimodal trained but unimodally retrieved. Composite bimodal memory was similar in strength to unimodal olfactory memory. For comparisons between groups with odor treatment, one-way ANOVA followed by Bonferroni correction and Tukey’s post-hoc test was done (∗p < 0.05, ∗∗∗p < 0.001). For comparisons between groups with visual treatment, Kruskal-Wallis test followed by Dunn-Bonferroni correction was done (∗∗∗p < 0.001). (C and D) Long-lasting memories retrieved after massed four-trial training plotted as changes in preference indices of trained and untrained flies to different combinations of unimodal or bimodal stimuli. Strengthening of olfactory long-lasting memory after 24 h was observed following bimodal training. Complete absence of visual long-lasting memory was also observed. For comparisons between normally distributed datasets, one-way ANOVA followed by Bonferroni correction and Tukey’s post-hoc test was done (∗p < 0.05, ∗∗∗p < 0.001). For comparisons between groups with visual treatment, Kruskal-Wallis test followed by Dunn-Bonferroni correction was done (∗∗∗p < 0.001).

Our initial experiments showed that flies exhibit a significant visual memory in a differential conditioning paradigm after four training trials (Figures 2E and 2F). In order to investigate whether the strength of the observed visual memory can be enhanced even further, we performed four-trial bimodal training experiments (i.e., 4x massed training) with an ITI of 1 min using one odor-color combination (AAC + green) (Figure 5A). Although we observed a trend of an increase when we trained the flies to a bimodal stimulus and retrieved only the visual memory, it was not significantly greater than unimodal visual memory, indicating that an upper limit might exist for the strength of learning the visual stimuli (Figure 5B). Additionally, similar to the previous experiments (Figures 4D and 4E), a significant decrease was observed in the strength of the olfactory memory when it was retrieved separately after four trials of differential bimodal training (Figure 5B). We also note here that the composite bimodal memory retrieved after bimodal training was not stronger but rather similar to the unimodal olfactory memory.

In experiments where testing immediately followed the one-trial training protocol, we observed that bimodal training had an incremental effect on weak/nonexistent visual learning (Figures 4B–4E). These results provide evidence for learning enhancement in at least one modality as a consequence of bimodal integration. However, the observation that we could not achieve a similar effect for olfactory learning led us to investigate potential underlying causes. We hypothesized that, while retrieved as a short-term memory, both one- and four-trial aversive olfactory training protocols achieved maximum learning efficiency, with little capacity for further memory enhancement. In addition, we also considered that enhancement following bimodal integration, if any, might not be prominently visible in our assay with such high unimodal learning scores. In order to test these hypotheses, we worked with a compromised or weakened olfactory memory that would provide us a window to observe an improvement. We performed experiments using the four-trial massed differential training protocol as described before but tested for a long-lasting memory after a period of 24 h (Figure 5A). Innate preferences to different stimuli were observed and indexed on the day of testing in different groups of flies. Two different combinations were used as CS+ (i.e., AAC + green or ETB + blue).

As expected, the unimodal long-lasting memory for both odors was weaker than the short-term memory that was obtained after the four-trial differential training protocol, with ETB retaining an even lower memory than AAC (Figures 5C and 5D). Notably, when bimodally trained but tested only with the odors after 24 h, flies exhibited a strengthened long-lasting olfactory memory for the odors. However, consistent with previous studies, we observed no visual long-lasting memory. This led us to conclude that despite not retaining any significant long-lasting memory of its own, just the presence of a visual stimulus during the training procedure leads to a stronger long-lasting olfactory memory. When compared to both the unimodal olfactory learning and short-term bimodal learning, we also observed a reduced composite bimodal memory after 24 h in case of AAC + green and nothing at all for ETB + blue. We attribute this observation to the severely compromised long-lasting memories of the visual and olfactory components.

## Discussion

We developed a modified T-maze that was adapted from the conventional Tully T-maze to perform aversive unimodal and bimodal learning experiments. Our setup consisted of simplified T-arms and the use of an improved odor-delivery system that ensured consistent odor concentration throughout the experimental duration (observed and quantified for up to 6 h). We used pulsed odor delivery that was synchronized exactly to the shock presentation, providing maximum temporal overlap between the stimuli (see STAR Methods). In addition, we employed LED panels to deliver the light stimulation in a homogeneous manner along the entire length of our training and testing tubes. The use of the same experimental apparatus to study both aversive olfactory and visual conditioning aided in studying the differences in learning efficiencies of the two sensory modalities. Significant learning scores were achieved after aversive visual training. Furthermore, the odors that we used in our study are food-derived compounds that elicit innate attraction in flies. The high learning scores that were observed for our chosen stimuli reaffirm the versatility of our assay.

### Learning and Memory Performances Vary for Distinct Stimuli, Both Within and Across Modalities

In our study, we demonstrated that identical training paradigms yield varying learning performances for different stimuli. *D. melanogaster* exhibits a very strong short-term aversive olfactory memory following a one-trial training paradigm. We show that repeating the same training cycle four times with an ITl of 1 min does not lead to enhancement of learning. It is also a noteworthy observation that after identical aversive training protocols, flies learned to avoid AAC and retained a stronger memory for it than for ETB. It is conceivable that different odors that hold different ecological meanings to the flies can be learned to different extents. In contrast to olfactory conditioning, the one-trial visual conditioning paradigm did not generate any significant learning performance in flies, and they required repeated reinforcements with four training trials to form a robust visual memory (Figures 2 and S2). Although significant visual learning after a single training trial was shown in an earlier study,^22^ the difference in our respective results could be attributed to a different testing protocol. In the abovementioned work, the US (i.e., 90V shock) was presented just before the testing phase for 1 s in order to arouse the flies after conditioning. This arousal may have a role in triggering more-efficient retrieval of the CS+ association after a single training trial. In our study, both blue and green wavelengths elicited similar learning responses in flies after 4x unimodal training. However, the effects of bimodal training were wavelength-specific for both short-term and long-lasting memory retrievals (Figures 4 and 5).

It is a clear advantage to learn and remember associations that are essential for survival in nature using the most reliable and precise sensory information. The ability of flies to learn olfactory cues more efficiently may therefore be due to their large dependence on chemosensation to perform diverse behaviors. Although shared circuits in the mushroom body underlie both visual and olfactory learning,^22^ we observe differences in the ease of acquisition, strength of learning, and memory retention between the two modalities, suggesting that intricate differences may still exist in the learning circuitry between them. The significant suppression of positive phototaxis after one-trial absolute conditioning can still be regarded as clear visual learning, while the same training protocol in a differential paradigm did not yield similar results. In the latter paradigm, flies required the training cycle to be repeated four times to show a significant aversive visual memory. It can be speculated that wavelength discrimination as a consequence of conditioning requires intense training trials, while the discrimination between the presence and absence of light (as in the case of absolute conditioning) can be learned and remembered more easily. These results indicate that visual learning, specifically discriminatory color learning, may not be employed by *D. melanogaster* in nature as extensively as odor learning to perform crucial behavioral decisions.

### Bimodal Integration Has Opposing Effects on Olfactory and Visual Memory Performance

Research involving bimodal sensory processing has proven in the past that cross-modal facilitation enhances innate as well as memory performances of the interacting sensory modalities.^41^^,^^42^^,^^43^^,^^44^^,^^45^ Our work suggests that such enhancement is conditional in the context of learning. Using wavelength discrimination as a form of weak visual learning, we corroborate the work done in the tethered flight arena, where subthreshold pattern learning of heat beams was enhanced by the addition of odor during the training.^32^^,^^46^ In our study, weak visual memory showed a clear improvement after bimodal training, specifically in experiments where unimodal visual training was unsuccessful (Figures 4B–4E). However, we also demonstrate that in a four-trial associative training, where an upper capacity for unimodal visual memory was already achieved, a significant enhancement could no longer be seen even after bimodal training (Figure 5B). Furthermore, we provide evidence that olfactory stimuli that are already strongly learned and memorized do not benefit from bimodal training. In fact, our results also indicate a reduction in the short-term olfactory memory when retrieved separately after bimodal training. We attribute this observation to the negative impact of a weakly learned visual stimulus on a strongly learned olfactory stimulus (Figures 4B–4E). Another possible reason for this reduction could be the removal of the exact training context during testing, which was previously shown to be important for a specific kind of long-term memory (LTM).^47^ However, we also observe a strengthened olfactory long-lasting memory after flies were bimodally trained but tested only with the odors and the visual context removed. This would imply that in our experiments, the context poses more weightage in the retrieval of the short-term memory than the long-lasting memory.

Previous studies in *Drosophila* have documented the formation of two long-lasting memory phases.^48^^,^^49^ A massed training protocol, as used here, is known to form a protein-synthesis-independent memory, also known as anesthesia-resistant memory (ARM).^50^ A spaced-training procedure, however, involves multiple training trials with a 15-min ITI and leads to the formation of protein-synthesis-dependent LTM. These memories also differ significantly in the underlying neuronal circuitry.^51^^,^^52^ In our study, we show strengthening of a weak, long-lasting olfactory memory retrieved 24 h after bimodal training. This could be either an enhancement effect or stabilization in the underlying circuit that slows down the decay of the memory trace and improves memory retrieval. While the unimodal olfactory long-lasting memory observed in our experiments resembles an ARM, it is conceivable that the memory formed after massed bimodal training may be consolidated differently. However, this mechanism is difficult to pinpoint without further experiments targeting the neuronal mechanisms that underlie memory consolidation after unimodal and bimodal training.

In our experiments, we observed no synergistic enhancement following bimodal training. In particular, our experiments with weakly learned stimuli such as ETB or blue color did not reveal the presence of any long-lasting bimodal memory. This implies that the inherent strength of unimodal memories could potentially contribute to the extent up to which bimodal training can affect them. Learned outcomes that cannot be associated or retrieved by one sensory modality might be supplemented by the presence of a different, strongly associated sensory modality. However, when the reinforcements are strong enough for the flies to sustain a unimodal memory association, the presence of an additional modality does not augment their learning anymore and can even cause detrimental effects if the additional modality is in itself weakly learned, calling into question the notion of “more the merrier.” The ability to learn and adapt to changes in the surroundings is crucial for all organisms not only to survive but also to thrive. Flies learn sensory signals to perform essential behaviors such as cost-efficient foraging trips, successful mating, and defense from natural enemies. In the context of conserving valuable costs during memory consolidation, it would serve the flies better to use the single, most reliable sensory stimulus to form important associations than to use multiple, but weakly learned, stimuli. Conversely, in cases where the ability to form robust unimodal associations is compromised, an additional cue reinforcing the same outcome can prove beneficial. We provide conclusive evidence that the effect of bimodal integration is not always synergistic but is conditional upon the ability of the flies to learn outcomes using information from the available modalities. The enhancement effect on visual memory after bimodal associative learning has also been shown to involve the odor-evoked activity of visually selective Kenyon cells in the mushroom bodies.^53^ However, the circuitry underlying the attenuation effect on inherently strong memories, as described in our study, is still unknown. Recent anatomical work done on the *D. melanogaster* brain connectome using electron microscopy techniques has revealed major new insights into the role of the lateral horn as a hub for multisensory inputs as well as associative learning.^54^^,^^55^^,^^56^ The neuronal substrates in the lateral horn and further downstream centers that may also play a role in these behaviors remain largely elusive so far and are therefore rich candidates for exploring the basis of multimodal integration.

### Limitations of the Study

Because our study focused on using optimized odor concentrations and light intensities that elicited an unbiased innate behavior, it is not possible to evaluate the effect of a range of concentrations or intensities, which may form unimodal memories of different strengths and, therefore, may also dictate how bimodally retrieved memories are consolidated. Furthermore, we only employed a limited choice of stimuli (i.e., two odors and colors). Lastly, although the T-maze provides a controlled arena to study learning behaviors, which can then be used to map neuronal circuitry, it may not reflect the real-time use of different stimuli by the insects in nature.

## STAR★Methods

### Key resources table

**Table:** 

REAGENT or RESOURCE	SOURCE	IDENTIFIER
**Chemicals, peptides, and recombinant proteins**		

Ethyl butyrate (99% pure)	Sigma Aldrich	CAS 105-54-4
Acetoin acetate (99% pure)	Sigma Aldrich	CAS 4906-24-5
Paraffin oil	Sigma Aldrich	CAS 8012-95-1

**Experimental models: Organisms/strains**		

Model organism: *Drosophila melanogaster*: Canton-S	Hansson lab strain	N/A
Model organism: *Drosophila melanogaster*: *w*^*1118*^*Ir8a*^*2*^*;+;Orco*^*2*^	Yael Grosjean lab strain	N/A

**Software and algorithms**		

OriginPro 2019 9.6.0.172	OriginLab Corporation	https://www.originlab.com
R software	R Core Team, 2020	https://www.r-project.org
GraphPad InStat 3	GraphPad Software Inc.	https://www.graphpad.com

**Other**		

Synchronized Stimulus Delivery System	This paper	N/A
LED Puzzles (452 nm, 520 nm, 720-30nm) 3W	Roschwege GmbH	https://www.roschwege.com/de/produkte/led-puzzle/
Siemens LOGO! PLC CPU 12V	Siemens, Munich, Germany	6ED1052-1MD08-0BA1

### Resource availability

#### Lead contact

Any requests for resources and information about methodology should be directed to and will be fulfilled by lead contact Silke Sachse (ssachse@ice.mpg.de).

#### Materials availability

This study did not generate new unique reagents. Information about design of the bimodal learning set-up with the synchronized stimulus delivery is available in the method details and upon request to the lead contact.

### Experimental model and subject details

*D.melanogaster* flies used for the behavioral assays throughout this study were reared in Corn-meal agar as a food medium at 25°C and 70% humidity in incubators manufactured by SnijdersLabs. 12:12 Light/Dark cycle was followed to preserve regular circadian rhythms. All behavioral experiments were performed with wild-type Canton-S flies, except in one experiment with olfactory mutants, where a double Orco^2^/Ir8a^2^ mutant was used. This line was a kind gift from Yael Grosjean and is mentioned in the key resources table. No sex-specific effects were seen, therefore 60-80 adults of both female and male sexes aged between 3 and 5 days post eclosion were used in the behavioral experiments. Each group of flies was used only once, either for determining innate preference or for the training protocol. After the completion of the experiments, flies were frozen and counted.

### Method details

#### Stimulus presentation in behavioral experiments

##### Continuous odor delivery

Two attractive odors were used in our experiments - acetoin acetate (AAC, CAS No. 4906-24-5, SIGMA) and ethyl butyrate (ETB, CAS No.105-54-4, SIGMA). For experiments specifically involving absolute conditioning, a continuous odor flow system was employed to provide flies with the odor stimuli. Odor dilutions in paraffin oil (PO) were standardized to observe sufficient attraction – AAC (≈10^−2^) and ETB (≈10^−3^). A flow rate of 0.35 L/min was used. These concentrations were observed to generate different levels of attraction depending on the type of odor delivery system used and the flow parameters.

##### Pulsated odor delivery

We adapted a new method of odor delivery for the differential conditioning experiments. Custom-modified 4 mL HPLC glass vials (MACHEREY-NAGEL GmbH & Co.KG) containing 1 mL of odorant were fitted with a metal capillary of a maximum diameter of 2.5 mm and were placed inside larger 250 mL bottles (Schott GmbH). Clean, compressed and humidified air was allowed to pass through these bottles at a constant and controlled flow rate of 0.1 L/min. The humidified odor headspace from the bottle was carried to the T-maze using Teflon tubes. Flow rates were controlled using digital flow meters (Flow sensors, FESTO). Volatile odor molecules in the glass vials evaporate into the headspace and an equilibrium of odor concentration is established and maintained with the headspace in the larger bottles. Standardization of the volatile measurement was done using Gas Chromatography coupled Mass Spectrometry (GC-MS). We observed that 1 h of odor accumulation in the larger bottle followed by 1 h of flushing with clean air was required to achieve consistent odor concentrations for a total pulsing duration of 6 h using the pure AAC and 1% ETB (solvent – paraffin oil, PO) in the glass vials. These concentrations elicited equal preference from the flies in a choice assay. Odor at a flow rate 0.35 L/min was delivered in pulses of 2 s followed by 2 s of only humidified air with 70% relative humidity (RH) maintained in the entire T-maze arena. Odor pulses were controlled with fast solenoid valves (Festo – Esslingen, Germany).

##### Light delivery

Custom made (3D-printed) contraptions with non-reflective black coating on the outside and a fitted LED panel on the inside (Roschwege, Greifenstein, Germany) of specific wavelengths - blue (452 nm) and green (520 nm) - were used to surround the entire surface of the testing and the training tubes to ensure homogeneous illumination of the arena. The training and testing tubes were made of transparent polymethyl methacrylate (PMMA). An intensity dimmer (HK Datentechnik, Dormagen, Germany) was fitted with the power supply for the LEDs (Meanwell, Taiwan) to standardize the optimal light intensities for the two wavelengths that ensured neutral innate preferences for the flies when given a choice.

##### Synchronization with the electric shock

The synchronization of the light stimulus, odor pulses and the electric shock was programmed using LOGO Direct Digital Control (DDC - Siemens LOGO, Munich, Germany). The odor pulses and the electric shock were synchronized to ensure sufficient temporal overlap and the light was presented uniformly across the duration of the protocol. A delay of approximately 200 ms was measured in the odor delivery and was taken into account while programming the synchronization.

#### Experimental protocols for aversive conditioning

##### Absolute conditioning

The T-maze was a custom-made 3D printed arena (polyoxymethylene material) adapted and simplified from the conventional Tully T-maze (Tully and Quinn, 1985). The arena was modified to ensure least interference from the reflected light (Figures 1A and 1B). Innate preference was determined by testing the choice of a group of naive flies (60–80 in number, 3–5 days old) to the odors and colors against paraffin oil and darkness, respectively. For the training protocol, a different group of 60–80 flies was aspirated into the training tube that was lined with an embedded copper grid (CON- Elektronik, Germany). Aversive reinforcement of the stimuli (CS+: odor/light/bimodal) was provided for 1 min paired with AC 90V electric shock. One training trial consisted of 15 shock pulses, each pulse lasting 1.8 s followed by an interval of 2.2 s. After training, flies were given 1 min of clean air or kept in darkness, then followed by 1 min of testing (odor vs paraffin oil/light vs dark). There was no electric shock presented during the testing. For bimodal training, CS + constituted a simultaneous presentation of the light, odor and shock. Reciprocal controls (PO + shock, dark + shock) were also performed to rule out non-associative effects (Figures 2A, 2B, and S1).

##### Differential conditioning

Innate preference of the flies to the odors (ETB vs AAC) and colors (blue vs green) was determined first in a separate group of naive flies. Since their respective concentrations and intensities were standardized, a neutral innate preference was ensured and therefore negated any inherent stimulus bias. A second group of naive flies was trained in a differential protocol. A single training trial consisted of 1 min of CS + paired with AC 90V electric shock. Flies were then given an interval of 30 s of clean air and darkness. This was followed by 1 min of CS- presentation. For a massed training protocol, these steps were repeated four times with an inter-trial interval (ITI) of 1 min. Testing was done for 1 min after the completion of the last CS- for 1 min (short-term memory) or for 90 s (massed training). There was no electric shock presented during the testing. The order of CS+ and CS- was not changed in between trials.

All experiments were conducted in the same time-window for all days to control for circadian variation in wavelength preferences. Suction was always provided to prevent odor contamination or accumulation in the center of the maze. The tube lined with the copper grid was used in both the training and the testing phase to reproduce the same contexts. All training and testing were done at 25°C and 70% relative humidity in a dark arena. Flies inside the arena were handled only in red light (>720 nm). Different groups of flies were used to observe innate and learned behavior. Preference indices were calculated for both using the following formula:

PreferenceIndex(PI)=[#Flies(CS+)−#Flies(CS−)]/[#Flies(CS+)+#Flies(CS−)]

In experiments where long-lasting memory retrieval was necessary, trained flies were collected and stored in vials with food at 25°C and darkness for 24 h.

### Quantification and statistical analysis

All statistical analyses and graphs were made using the software OriginPro2019 9.6.0.172 (OriginLab corporation). At a significance level of 0.05, datasets were checked for normal distribution using the Shapiro-Wilk test. When normality was established, comparisons between the groups were done using One-Way ANOVA followed by either Bonferroni correction or a post-hoc Tukey’s test. For data with nonparametric distribution, Kruskal-Wallis test followed by a post-hoc Dunn- Bonferroni tests were carried out. Comparisons between two normally distributed groups were done using unpaired two-tailed Student T-test. The significance level was set at 0.05 and is denoted by asterisks (∗p < 0.05, ∗∗p < 0.01, ∗∗∗p < 0.001).

## Data Availability

• All raw data reported in this paper will be shared by the lead contact upon request. • This paper does not report original code. • Any additional information required to reanalyze the data reported in this paper is available from the lead contact upon request.
