# Supplementary material for: Aversive Bimodal Associations Differently Impact Visual and Olfactory Memory Performance in Drosophila
Source: iScience. 2022 Nov 3;25(12):105485. doi: 10.1016/j.isci.2022.105485 (PMC9672954; doi:10.1016/j.isci.2022.105485)
Supplement: Document S1. Figures S1 and S2 [file mmc1.pdf]

iScience, Volume *25*

Supplemental information

Aversive Bimodal Associations Differently Impact Visual and Olfactory Memory Performance in *Drosophila*

Devasena Thiagarajan, Franziska Eberl, Daniel Veit, Bill S. Hansson, Markus Knaden, and Silke Sachse

FIGURE S1

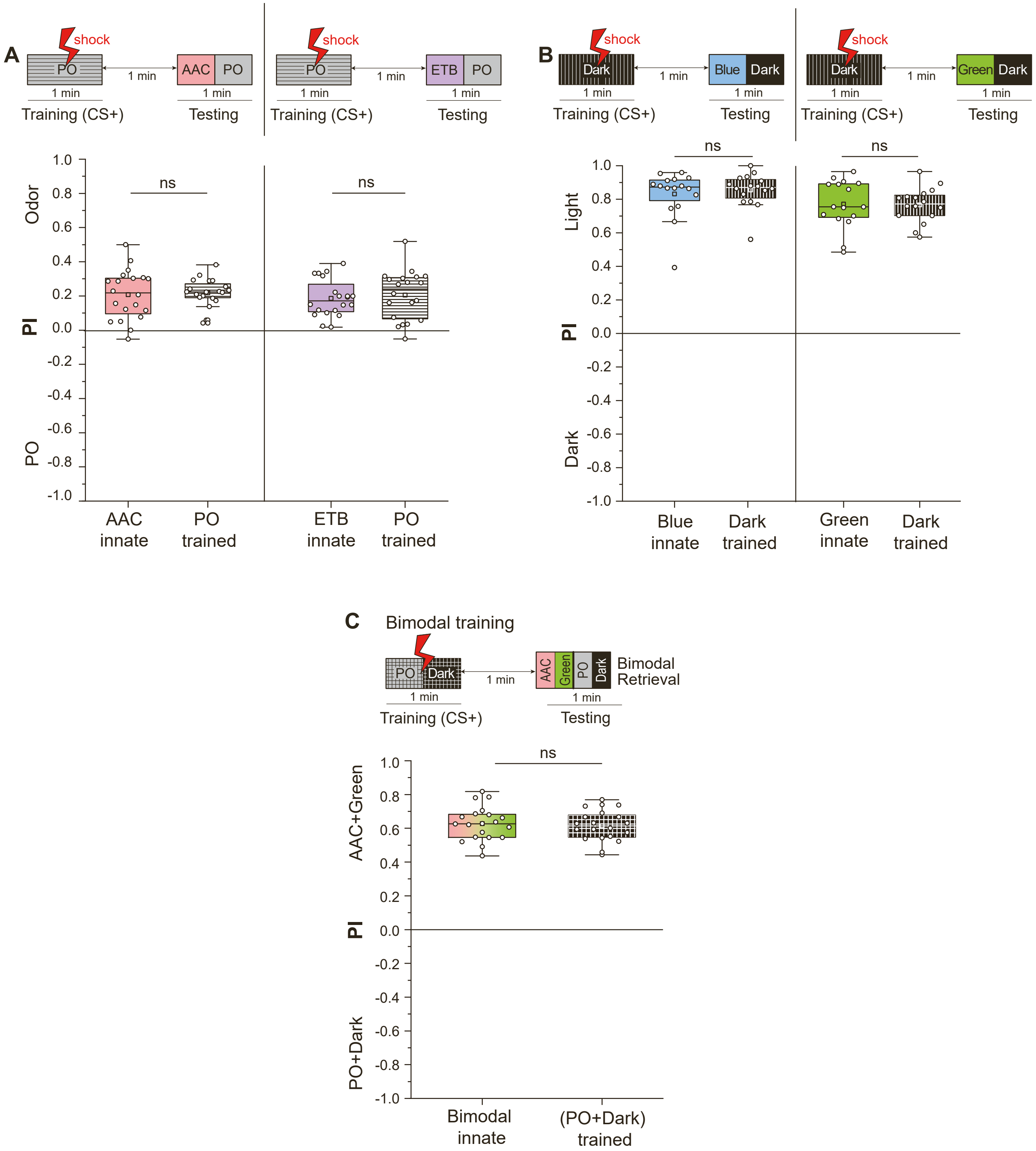

A
shock
PO
1 min
AAC
PO
1 min
Training (CS+)
Testing
shock
PO
1 min
ETB
PO
1 min
Training (CS+)
Testing
Odor
PI
PO
ns
ns
1.0
0.8
0.6
0.4
0.2
0.0
-0.2
-0.4
-0.6
-0.8
-1.0
AAC innate
PO trained
ETB innate
PO trained
B
shock
Dark
1 min
Blue
Dark
1 min
Training (CS+)
Testing
shock
Dark
1 min
Green
Dark
1 min
Training (CS+)
Testing
Light
PI
Dark
ns
ns
Blue innate
Dark trained
Green innate
Dark trained
C
Bimodal training
PO
Dark
1 min
AAC
Green
PO
Dark
Bimodal Retrieval
1 min
Training (CS+)
Testing
AAC+Green
PI
PO+Dark
ns
Bimodal innate
(PO+Dark) trained

FIGURE S2

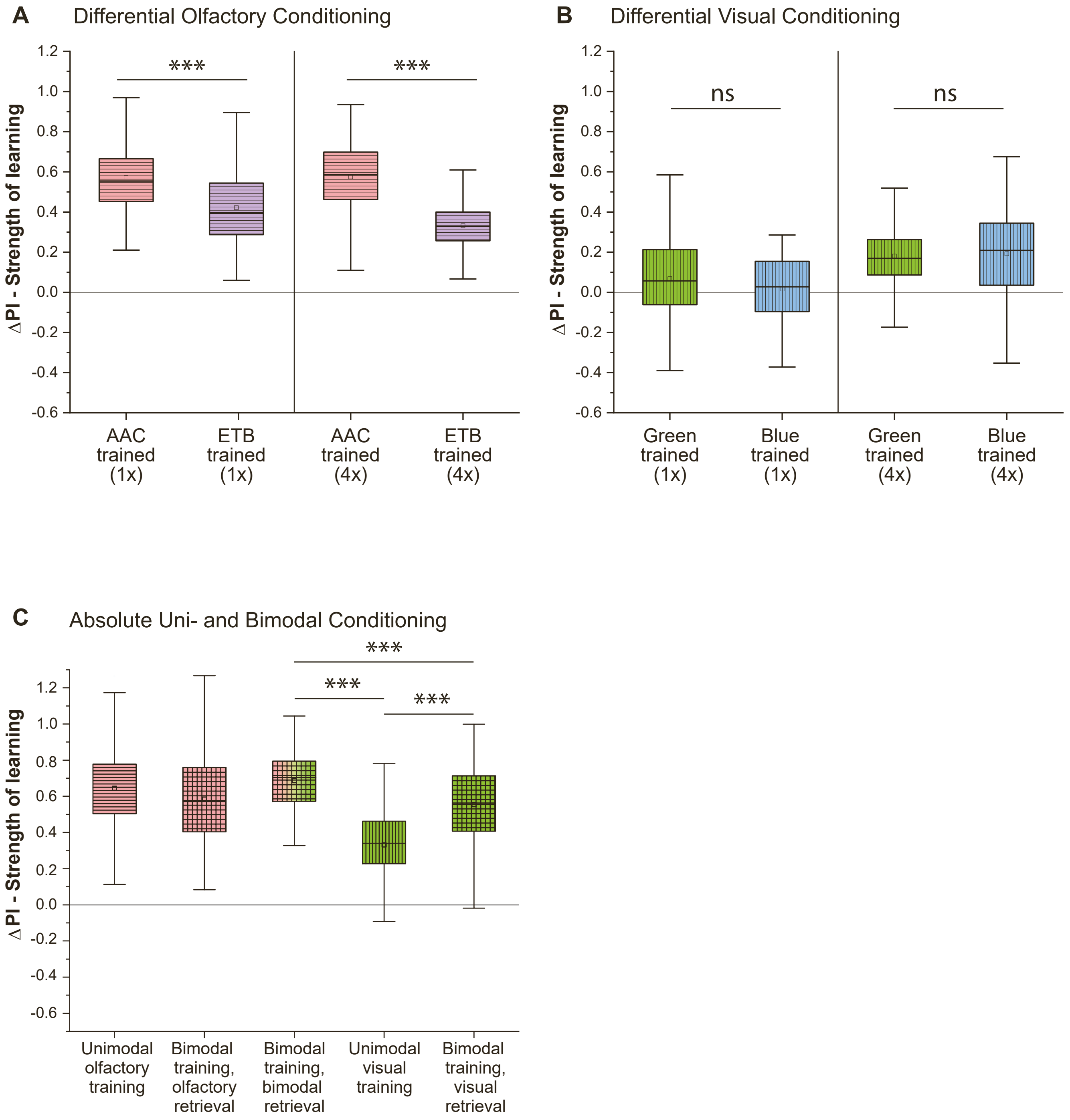

A
Differential Olfactory Conditioning
ΔPI - Strength of learning
1.2
1.0
0.8
0.6
0.4
0.2
0.0
-0.2
-0.4
-0.6
***
***
AAC trained (1x)
ETB trained (1x)
AAC trained (4x)
ETB trained (4x)
B
Differential Visual Conditioning
ns
ns
Green trained (1x)
Blue trained (1x)
Green trained (4x)
Blue trained (4x)
C
Absolute Uni- and Bimodal Conditioning
***
***
***
Unimodal olfactory training
Bimodal training, olfactory retrieval
Bimodal training, bimodal retrieval
Unimodal visual training
Bimodal training, visual retrieval

Supplemental Figure Legends

Figure S1. Non-associative controls for the absolute conditioning experiments (related to Figures 2A, B and 3D). A. Protocol depicting the training of flies to associate paraffin oil (PO) with an electric shock followed by testing their absolute choices to acetoin acetate (AAC) or ethyl butyrate (ETB) against PO. Preference indices plotted for flies that were aversively trained to PO did not differ significantly from those of naïve flies. **B.** Protocol depicting the training of flies to associate darkness with an electric shock followed by testing their absolute choices to blue or green wavelengths against darkness. Preference indices plotted for flies that were aversively trained to darkness did not differ significantly from those of naïve flies. **C.** Similar protocol and preference index plotted for absolute bimodal training with PO+dark. These results control for any non-associative effects. Comparison between two normally distributed data sets was made using unpaired Student T-test with a Welch's correction (ns - $p>0.05$).

Figure S2. ∆PI values depicting strength of learning across unimodal and bimodal training paradigms (related to Figures 2C-F and 3D). Pairwise differences for all possible combinations were calculated between the innate and learned preference indices of different odors and colors, depicting the strength of learning. **A.** ∆PI values for AAC and ETB indicate significant differences in the ability of flies to learn the two odors. **B.** ∆PI values for blue and green training, which are not significantly different. **C.** ∆PI values for absolute bimodal conditioning experiments (as shown in Fig. 3D), with significant difference evident between unimodally and bimodally acquired visual memory. Comparison between two normally distributed data sets was made using unpaired Student T-test with a Welch's correction (***$p<0.001$). For comparisons between normally distributed datasets, one-way ANOVA followed by Bonferroni correction and Tukey's post-hoc test was done (*$p<0.05$, ***$p<0.001$).
